# Multi-location gram-positive and gram-negative bacterial protein subcellular localization using gene ontology and multi-label classifier ensemble

**DOI:** 10.1186/1471-2105-16-S12-S1

**Published:** 2015-08-25

**Authors:** Xiao Wang, Jun Zhang, Guo-Zheng Li

**Affiliations:** 1 School of Computer and Communication Engineering, Zhengzhou University of Light Industry, Zhengzhou, China; 2 The Key Laboratory of Embedded System and Service Computing, Ministry of Education, Department of Control Science and Engineering, Tongji University, Shanghai, China; 3 Information Management Center, Training Department, Beijing Armed Police Command College, Beijing, China

**Keywords:** multi-location proteins, subcellular localization, gene ontology, multi-label classifier ensemble

## Abstract

**Background:**

It has become a very important and full of challenge task to predict bacterial protein subcellular locations using computational methods. Although there exist a lot of prediction methods for bacterial proteins, the majority of these methods can only deal with single-location proteins. But unfortunately many multi-location proteins are located in the bacterial cells. Moreover, multi-location proteins have special biological functions capable of helping the development of new drugs. So it is necessary to develop new computational methods for accurately predicting subcellular locations of multi-location bacterial proteins.

**Results:**

In this article, two efficient multi-label predictors, Gpos-ECC-mPLoc and Gneg-ECC-mPLoc, are developed to predict the subcellular locations of multi-label gram-positive and gram-negative bacterial proteins respectively. The two multi-label predictors construct the GO vectors by using the GO terms of homologous proteins of query proteins and then adopt a powerful multi-label ensemble classifier to make the final multi-label prediction. The two multi-label predictors have the following advantages: (1) they improve the prediction performance of multi-label proteins by taking the correlations among different labels into account; (2) they ensemble multiple CC classifiers and further generate better prediction results by ensemble learning; and (3) they construct the GO vectors by using the frequency of occurrences of GO terms in the typical homologous set instead of using 0/1 values. Experimental results show that Gpos-ECC-mPLoc and Gneg-ECC-mPLoc can efficiently predict the subcellular locations of multi-label gram-positive and gram-negative bacterial proteins respectively.

**Conclusions:**

Gpos-ECC-mPLoc and Gneg-ECC-mPLoc can efficiently improve prediction accuracy of subcellular localization of multi-location gram-positive and gram-negative bacterial proteins respectively. The online web servers for Gpos-ECC-mPLoc and Gneg-ECC-mPLoc predictors are freely accessible at http://biomed.zzuli.edu.cn/bioinfo/gpos-ecc-mploc/ and http://biomed.zzuli.edu.cn/bioinfo/gneg-ecc-mploc/ respectively.

## Background

Bacteria widely distributed in soil and water, or coexistence with other creatures, which are the most one in all organisms. All bacteria are grouped into prokaryotes that have a very simple cell structure lacking a cell nucleus, mitochondria and chloroplasts. Bacteria can be classified into two groups via Gram staining method: Gram-positive and Gram-negative. The former are stained dark blue or violet by Gram staining, while the latter instead appear red or pink. Because the functions of proteins are closely related to their subcellular locations, knowing subcellular locations of proteins in a bacterial cell can help biologists elucidating the functions of proteins and thus screening candidates in drug design.

Nowadays, there are two methods for identifying the subcellular locations of proteins: biochemical experiments and computational methods. In the post-genomic era, with the completion of various sequencing projects, new protein sequences have grown exponentially [1]. The biochemical experiments not only consume a lot of time but also pay high costs, and thus they have not adapted to the new situation. It is required to develop computational methods to identify the subcellular locations of these proteins automatically and accurately.

Computational methods for protein subcellular localization prediction can be roughly divided into the following four groups: (1) sequence-based methods; (2) sorting-signals based methods; (3) homology-based methods and (4) annotation-based methods. Sequence-based methods include, such as amino acid compositions (AAC) [2-4], amino acid pair compositions or dipeptide compositions [5,6], gapped amino acid pair compositions [5,7], and pseudo amino acid composition (PseAAC) [8-10]; sorting-signals based methods, such as PSORT [11], WoLF PSORT [12], TargetP [13] and SignalP [14,15]; homology-based methods, such as Proteome Analyst [16] and PairProSVM [17]; annotation-based methods, such as MultiLoc2 [18], SherLoc2 [19], Hum-PLoc [20], Gneg-PLoc [21], iLoc-Hum [22], ProLoc-GO [23].

Although there exist a lot of prediction methods for subcellular localization of proteins, the majority of these methods can only deal with single-location proteins. But unfortunately many multi-location proteins are located at more than one location site simultaneously. When prediction models are constructed by these methods, multi-location proteins are not included in the training set. Actually, multi-location proteins have special biological functions capable of helping the development of new drugs.

There are only a few predictors [21,24-32] specifically developed for predicting gram-positive and gram-negative bacterial proteins. To the best of our knowledge, there are only four predictors, namely Gpos-mPLoc [31], iLoc-Gpos [30], Gneg-mPLoc [26] and iLoc-Gneg [32], capable of predicting multi-label gram-positive and gram-negative bacterial proteins. iLoc-Gpos and iLoc-Gneg perform better than Gpos-mPLoc and Gneg-mPLoc respectively because the formers propose a better prediction algorithm to identity sub-cellular locations of query proteins.

In this article, two efficient multi-label predictors, Gpos-ECC-mPLoc and Gneg-ECC-mPLoc, are proposed to predict the subcellular locations of multi-label gram-positive and gram-negative bacterial proteins respectively. The two multi-label predictors extract GO feature vectors from GO terms of homologs of query proteins and then adopt a powerful multi-label ensemble classifier to output the final multi-label prediction results. Experimental results show that Gpos-ECC-mPLoc and Gneg-ECC-mPLoc can efficiently predict the subcellular locations of multi-label gram-positive and gram-negative bacterial proteins respectively. For readers'convenience, we developed the online web servers for Gpos-ECC-mPLoc and Gneg-ECC-mPLoc predictors which are freely accessible at http://biomed.zzuli.edu.cn/bioinfo/gpos-ecc-mploc/ and http://biomed.zzuli.edu.cn/bioinfo/gneg-ecc-mploc/ respectively.

## Results and discussion

### Datasets

In this article, the gram-positive bacterial benchmark dataset used in Gpos-mPLoc [31] and iLoc-Gpos [30] and the gram-negative bacterial benchmark dataset used in Gneg-mPLoc [26] and iLoc-Gneg [32] are utilized to evaluate the prediction performance of Gpos-ECC-mPLoc and Gneg-ECC-mPLoc respectively.

The gram-positive bacterial dataset consists of 519 gram-positive bacterial proteins, which are distributed in 4 locations (see Table 1). Of the 519 gram-positive bacterial proteins, 515 belong to one subcellular location, 4 to two locations, and none to more locations. The number of locative proteins in this dataset is 523. The concept of locative proteins and actual proteins have been explained in detail in literature [33-35]. The sequence identity in this dataset is controlled fewer than 25%.

**Table 1:** Breakdown of the gram-positive bacterial benchmark dataset.

Order	Subcellular location	Number of proteins
1	Cell membrane	174
2	Cell wall	18
3	Cytoplasm	208
4	Extracell	123
Total number of locative proteins		523
Total number of different proteins		519

The gram-negative bacterial dataset consists of 1392 gram-negative bacterial proteins, which are distributed in 8 locations (see Table 2). Of the 1392 gram-negative bacterial proteins, 1328 belong to one subcellular location, 64 to two locations, and none to more locations. The number of locative proteins in this dataset is 1456. The sequence identity in this dataset is also controlled fewer than 25%.

**Table 2:** Breakdown of the gram-negative bacterial benchmark dataset.

Order	Subcellular location	Number of proteins
1	Cell inner membrane	557
2	Cell outer membrane	124
3	Cytoplasm	410
4	Extracellular	133
5	Fimbrium	32
6	Flagellum	12
7	Nucleoid	8
8	Periplasm	180
Total number of locative proteins		1456
Total number of different proteins		1392

### Performance measures

In this article, we use the (overall) locative and absolute accuracy to measure the performance of multi-label predictors. The overall locative and absolute accuracy are defined as follows:

(1) $$\text{overall locative accuracy}=\frac{1}{N_{loc}}\overset{N_{di\;f}}{\underset{i=1}{\sum }}\left|Y_{i}\cap Z_{i}\right|$$

(2) $$\text{overall absolute accuracy}=\frac{1}{N_{di\;f}}\overset{N_{di\;f}}{\underset{i=1}{\sum }}\;1\;\left(Y_{i}\equiv Z_{i}\right)$$

where *Y_i _*is the set of true labels of each protein, *Z_i _*the set of predicted labels of each one, *N_loc _*the number of locative proteins, *N_dif _*the number of different proteins, *| *- *| *the operator acting on the set to count the number of its elements, *∩ *the intersection of sets, 1(*Y_i _≡ Z_i_*) equals 1 if true labels are entirely identical to predicted labels, 0 otherwise.

When and only when all of the subcellular locations of a query protein are exactly predicted, the prediction result of the query protein can be considered as correct. Therefore, the overall absolute accuracy is stricter than the overall locative accuracy. For the two measures, more detailed explanation can be found in [36].

### Comparison with the state-of-the-art predictors

In statistical prediction, the jackknife test, also named leave-one-out cross validation, is considered as the most rigorous and objective evaluation method [37]. The jackknife test has been widely utilized by researchers to evaluate the performance of various prediction methods [38-43]. Hence, in this article, we also use the jackknife test to evaluate the prediction performance of our proposed Gpos-ECC-mPLoc and Gneg-ECC-mPLoc predictors.

For the Gpos-ECC-mPLoc predictor, we compare our proposed Gpos-ECC-mPLoc predictor with two state-of-the-art gram-positive bacterial multi-label predictors, i.e., Gpos-mPLoc [31] and iLoc-Gpos [30] predictors. For the Gneg-ECC-mPLoc predictor, we also compare our proposed Gneg-ECC-mPLoc predictor with two state-of-the-art gram-negative bacterial multi-label predictors, i.e., Gneg-mPLoc [26] and iLoc-Gneg [32] predictors. Ensemble sizes of multi-label ensemble classifiers (i.e., ECC) used in Gpos-ECC-mPLoc and Gneg-ECC-mPLoc are respectively set to 25 and 40 for achieving the best performance.

Table 3 shows the comparison results of our proposed Gpos-ECC-mPLoc predictor against two state-of-the-art gram-positive bacterial multi-label predictors on the gram-positive bacterial benchmark dataset by the jackknife test. Similar to both Gpos-mPLoc [31] and iLoc-Gpos [30], Gpos-ECC-mPLoc also uses the accession numbers of homologous proteins of query proteins to retrieve corresponding GO terms from the GOA database. Gpos-ECC-mPLoc utilizes homologous proteins which have *≥ *60% pairwise sequence similarity with the query protein. Note that if a query protein do not have any homologous protein or accession numbers of its homologous proteins do not match any GO term from the GOA database, dipeptide composition method is used as a backup for extracting its feature vector. In the gram-positive bacterial benchmark dataset, there is one protein without any homologs.

**Table 3:** Performance comparison of Gpos-ECC-mPLoc with the state-of-the-art predictors on the gram-positive bacterial benchmark dataset by the jackknife test.

Order	Subcellular location	Success rate by jackknife test		
		
		Gpos-ECC-mPLoc	Gpos-mPLoc	iLoc-Gpos
1	Cell membrane	96.53%	-	95.98%
2	Cell wall	66.67%	-	66.67%
3	Cytoplasm	96.15%	-	95.19%
4	Extracell	92.68%	-	89.43%
Overall locative accuracy		94.44%	82.2%	93.12%
Overall absolute accuracy		94.02%	-	92.87%

Table 4 shows the comparison results of our proposed Gneg-ECC-mPLoc predictor against two state-of-the-art gram-negative bacterial multi-label predictors on the gram-negative bacterial benchmark dataset by the jackknife test. Gneg-mPLoc [26] uses similar methods as Gpos-mPLoc [31], and iLoc-Gneg [32] uses similar methods as iLoc-Gpos [30]. Gneg-ECC-mPLoc also utilizes homologous proteins which have *≥ *60% pairwise sequence similarity with the query protein. In the gram-negative bacterial benchmark dataset, there are two proteins without any homologs.

**Table 4:** Performance comparison of Gneg-ECC-mPLoc with the state-of-the-art predictors on the gram-negative bacterial benchmark dataset by the jackknife test.

Order	Subcellular location	Success rate by jackknife test		
		
		Gneg-ECC-mPLoc	Gneg-mPLoc	iLoc-Gneg
1	Cell inner membrane	95.5%	94.3%	96.8%
2	Cell outer membrane	94.4%	84.7%	83.1%
3	Cytoplasm	92.2%	87.1%	89.5%
4	Extracellular	93.2%	59.4%	86.5%
5	Fimbrium	93.8%	87.5%	93.8%
6	Flagellum	100%	0.0%	100%
7	Nucleoid	87.5%	0.0%	50%
8	Periplasm	94.4%	85.6%	89.4%
Overall locative accuracy		94.1%	85.7%	91.4%
Overall absolute accuracy		92.4%	-	89.9%

As can be seen from Table 3 and 4, for the gram-positive bacterial dataset, Gpos-ECC-mPLoc performs better than Gpos-mPLoc and iLoc-Gpos; for the gram-negative bacterial dataset, Gneg-ECC-mPLoc also performs better than Gneg-mPLoc and iLoc-Gneg. Specifically, in the gram-positive bacterial dataset, the overall locative accuracy achieved by Gpos-ECC-mPLoc is 94.44%, which is more than 12% higher than that achieved by Gpos-mPLoc and 1% higher than that achieved by iLoc-Gpos, while the overall absolute accuracy of Gpos-ECC-mPLoc is 94.02%, which is more than 1% higher than iLoc-Gpos; and in the gram-negative bacterial dataset, Gneg-ECC-mPLoc achieves 94.1% overall locative accuracy, with more than 8% performance improvement against Gneg-mPLoc and approximately 3% improvement against iLoc-Gneg, while Gneg-ECC-mPLoc achieves 92.4% overall absolute accuracy, with approximately 3% improvement against iLoc-Gneg. The results on both datasets show that Gpos-ECC-mPLoc and Gneg-ECC-mPLoc are more capable of handling multi-label problems than Gpos-mPLoc, iLoc-Gpos, Gneg-mPLoc and iLoc-Gneg. That is because Gpos-ECC-mPLoc and Gneg-ECC-mPLoc take correlations among subcellular locations into account, while Gpos-mPLoc, iLoc-Gpos, Gneg-mPLoc and iLoc-Gneg only transform the multi-label classification problem to one single-label classification problem and thus lose the beneficial label correlations information. Moreover, ensembling multiple multi-label classifiers in Gpos-ECC-mPLoc and Gneg-ECC-mPLoc further enhances the prediction performance. As for the individual locative accuracy, in the gram-positive bacterial dataset, Gpos-ECC-mPLoc achieves the similar locative accuracies to iLoc-Gpos for the 'Cell membrane', 'Cell wall' and 'Cytoplasm', while the locative accuracy of Gpos-ECC-mPLoc is remarkably higher than iLoc-Gpos for the 'Extracell'; in the gram-negative bacterial dataset, the locative accuracies of Gneg-ECC-mPLoc for all of the 8 locations are significantly higher than Gneg-mPLoc, except for the 'Cell inner membrane', 'Fimbrium' and 'Flagellum' for which both Gneg-ECC-mPLoc and iLoc-Gneg achieve the similar locative accuracies, while Gneg-ECC-mPLoc performs remarkably better than iLoc-Gneg for the rest of location sites.

## Conclusions

In this article, we propose two efficient multi-label predictors, Gpos-ECC-mPLoc and Gneg-ECC-mPLoc, to predict the subcellular locations of multi-label gram-positive and gram-negative bacterial proteins respectively. The two multi-label predictors use the GO terms of homologous proteins of query proteins to construct the GO vectors and then the GO vectors are fed into the powerful ensemble of classifier chains (ECC) classifier for generating the final multi-label prediction results. Compared with the existing predictors, Gpos-ECC-mPLoc and Gneg-ECC-mPLoc have three following advantages: (1) CC takes the correlations among different labels into account and then improves the prediction performance of multi-label proteins; (2) ECC ensembles multiple CC classifiers and can generate better prediction results by ensemble learning; and (3) they construct the GO vectors by using the frequency of occurrences of GO terms in the typical homologous set instead of using 0/1 values.

Experimental results show that Gpos-ECC-mPLoc and Gneg-ECC-mPLoc can efficiently predict the subcellular locations of multi-label gram-positive and gram-negative bacterial proteins respectively. For readers'convenience, the online web servers for Gpos-ECC-mPLoc and Gneg-ECC-mPLoc predictors are freely accessible at http://biomed.zzuli.edu.cn/bioinfo/gpos-ecc-mploc/ and http://biomed.zzuli.edu.cn/bioinfo/gneg-ecc-mploc/ respectively.

## Methods

### Feature extraction

#### Gene ontology

The Gpos-ECC-mPLoc and Gneg-ECC-mPLoc predictors only use amino acid sequences as input and do not need to know the accession numbers of query proteins in advance. Given a query protein, its amino acid sequence is entered to BLAST [44] to search its homologous proteins. Those homologous proteins with *≥ *60% pairwise similarity are picked out as the typical homologous set of the query protein. Corresponding GO terms of the query protein are retrieved from the GOA database using the accession numbers of its typical homologous set as the keys. Note that for a different query protein, the number of its typical homologous set may be different.

In this article, we used the GOA database released on 08-Apr-2011, which consists of 18844 distinct GO terms. These GO terms form an Euclidean space with 18844 dimensions. Given a dataset, we used the procedure described in the above to retrieve the GO terms of all of its proteins. For each protein in the dataset, it can be represented as a GO vector by matching its GO terms to all of the 18844 GO terms. We used the approach described in [45,46] to determine the elements of the GO vectors. Specifically, the GO vector *p_i _*of the i-th protein is defined as:

(3) $$p_{i}=\left[\begin{array}{c} f_{i,\;1}\\ \vdots \\ f_{i,\;j}\\ \vdots \\ f_{i,\;18844} \end{array}\right]\;\text{where}\;f_{i,\;j}=\frac{\sum_{k=1}^{N_{h}}g\;\left(j,\;k\right)}{N_{h}}$$

where *N_h _*is the number of its typical homologous set, *g *(*j, k*) = 1 if the k-th homologous protein hits the j-th GO term, *g *(*j, k*) = 0 otherwise, and *f_i,j _*means the frequency of occurrences of the j-th GO term in the typical homologous set.

#### Dipeptide composition

Some proteins can not be represented as GO vectors because they do not have any homologous proteins or accession numbers of their homologous proteins do not match any GO term from the GOA database. In this article, dipeptide composition is used as a backup, which represents the frequency of occurrences of each two adjacent amino acid residues. 420-dimensional vectors are generated by the dipeptide composition for the query proteins, in which the first 20 elements are the conventional amino acid composition (AAC), the following 400 elements are the frequency of occurrences of the 400 different dipeptides.

### Prediction method

#### Binary relevance

Binary relevance method (BR) [47] uses the one-against-rest strategy to convert a multi-label problem into several binary classification problems. Given a multi-label dataset with N class labels, BR method trains one classifier for each class label. When training one classifier for each class label, BR method annotates all of the training examples associated with that label as positive examples while all remaining examples are regarded as negative examples. Given a test example, each classifier in BR will output a prediction score and BR will combine these scores into a N-dimensional score vector, where each score corresponds to a specific class label. The value of the score has two conditions, positive and negative, positive means the binary classifier predicts the test example belongs to the corresponding class label, negative means it do not belong to the class label. Note that if all N scores are negative, the class label with the maximum score is assigned to the test example.

#### Classifier chain

Classifier Chain (CC) method [48] is derived from BR method and also makes up of N binary classifiers as in BR. Unlike BR, each classifier in CC has to be trained sequentially. Classifiers in CC are then linked along a chain in sequence that they are trained. Because examples in a multi-label dataset could have multiple class labels and class labels may be correlated, CC thus takes the correlations among class labels into account. It extends the feature space of each classifier in the chain with the predicted labels of all previous classifiers. Since CC method passes class label information between classifiers, CC takes label correlations into account and thus overcomes the label independence weakness of BR method. The process of making the prediction in the CC method is the same as in the BR method.

#### Ensemble of classifier chains

Considering an ensemble of multiple classifiers generally generates a better prediction accuracy [49], we construct an multi-label classifier ensemble by combining multiple CC classifiers. Because different label orders could generate different prediction results, ensemble of classifier chains (ECC) trains multiple different CC classifiers, where each CC classifier is trained with a random chain order. Each CC classifier will outputs a score vector, we then take the average of these score vectors to make the final predictions by the prediction process as described in the BR method. In this article, we use ECC as the prediction engine in Gpos-ECC-mPLoc and Gneg-ECC-mPLoc.

#### Support vector machine

Each classifier in BR and CC method can be trained by different binary classification algorithm. For simplicity, in this article, we use support vector machine (SVM) [50] as the base learner to train each classifier in CC method. SVM is a well-known binary classification algorithm and commonly used in various fields of bioinformatics [28,51-57]. The LIBLINEAR software package [58] is used to train SVM. It is very efficient and designed specially for high dimensional vectors as the GO vectors used in this work.

## Competing interests

The authors declare that they have no competing interests.

## Authors' contributions

XW and GZL conceived the idea of this article. XW designed the experiments. JZ performed the experiments. XW analyzed the data and wrote the article. GZL supervised the whole work. All authors read and approved the final manuscript.

## Declarations

The publication costs for this article were partly funded by grants from National Natural Science Foundation of China (61402422 and 61273305), Key Project of Science and Technology Research of the Education Department of Henan Province (14A520063), Doctoral Research Fund of Zhengzhou University of Light Industry (2013BSJJ082) and Open Fund of MOE Key Laboratory of Embedded System and Service Computing of Tongji University (ESSCKF201308).

This article has been published as part of *BMC Bioinformatics *Volume 16 Supplement 12, 2015: Selected articles from the IEE International Conference on Bioinformatics and Biomedicine (BIBM 2014): Bioinformatics. The full contents of the supplement are available online at http://www.biomedcentral.com/bmcbioinformatics/supplements/16/S12.
